# A mixed-method evaluation of the views of medical teachers on the applicability of the ‘infant and young child feeding chapter’ in Saudi medical colleges

**DOI:** 10.1186/s12909-018-1282-3

**Published:** 2018-10-08

**Authors:** Fouzia Al-Hreashy, Hanan Al-Kadri, Abduelah Al-Mobeirek, Albert Scherpbier

**Affiliations:** 1 Department of Family Medicine, College of Medicine, Al-Imam Mohammad Ibn Saud Islamic University (IMSIU), PO BOX 62510, Riyadh, 11595 Saudi Arabia; 2 Department of Obstetrics and Gynecology, College of Medicine, King Saud Bin Abdulaziz University for Health Sciences, Riyadh, Saudi Arabia; 3 Department of Cardiology, King Saud University, Riyadh, Saudi Arabia; 4 Department of Educational Development and Research, School of Health Professions Education, Faculty of Health, Medicine and Life Sciences, Maastricht University, Maastricht, The Netherlands

**Keywords:** Breastfeeding, Infant nutrition, Lactation, Education, Curriculum, Medicine, Saudi Arabia

## Abstract

**Background:**

Lack of sufficient preparation of physicians for the provision of breastfeeding support and counselling has been well-documented. The development of training in breastfeeding medicine for medical students is currently ongoing worldwide. This study was conducted to gain insights into a potential framework for a breastfeeding education curriculum.

**Methods:**

A mixed-method design was used to evaluate the opinions of medical teachers regarding current lactation education and the applicability of the World Health Organization ‘Infant and young child feeding: model chapter for textbooks for medical students and allied health professionals’ in medical colleges in Riyadh, Saudi Arabia. Twelve teachers from three medical schools were invited to participate in three rounds of research. The first round was carried out through an interview using open-ended questions under three headings: 1) The general opinion on breastfeeding medicine education in medical colleges; 2) The opinion on the contents of the chapter under investigation; and 3) The opinion on cultural points regarding Saudi Arabia and breastfeeding education in medical colleges. This was followed by a thematic analysis. Self-administered, closed-ended questionnaires were created for the second round based the results of the first round. The third round addressed areas of disagreement in opinions. To assess the degree of agreement objectively, rounds 2 and 3 were analyzed according to the 5-point Likert scale, with responses merged to a 3-point Likert scale where appropriate. A consensus was reached when greater than 70% agreement achieved.

**Results:**

All participants agreed that breastfeeding education is suboptimal. Although they considered the world health organization resource on infant and young child chapter a suitable reference for the curriculum, they agreed that modifications to suit the Saudi Arabian context are necessary. The medical teachers suggested a unique curriculum for medical students, which is similar for both genders. However, disagreement existed regarding the provision of extra clinical training to female students.

**Conclusions:**

Breastfeeding medicine education in medical colleges should be developed using resources that are rich in content, are physician-specific and take into consideration the culture.

## Background

The curricula of medical schools are designed to graduate clinically competent medical students. Major changes are ongoing in both the content and process of medical education. For example, vertical integration of basic science, clinical studies and community-based learning are common in contemporary medical education [1]. The Saudi framework for medical schools encourages the introduction of health promotion and disease prevention in the undergraduate medical curriculum [2]. At the same time, changes in the medical curriculum that address maternal and child health issues have reduced infant mortality in developing countries [3]. Breastfeeding is the normative standard for infant feeding and nutrition. Given the documented short- and long-term advantages of breastfeeding, infant nutrition should be considered a public health concern [4], particularly in relation to the trend toward decreased breastfeeding in Saudi Arabia [5, 6]. The Academy of Breastfeeding Medicine describes the physician’s role in breastfeeding promotion and recommends breastfeeding education to clinicians, starting in the preclinical phase of training [7].

Lack of sufficient preparation of physicians for providing breastfeeding support and counselling has been well-documented in the medical literature [8–12]. Furthermore, the decision-making process by physicians regarding medication use during breastfeeding lacks a negotiated or evidence-based approach among health care professionals [13, 14]. Even mothers with experience in the medical professions often cannot overcome difficulties with breastfeeding. Women in medicine need enhanced breastfeeding education, support, services and resources. One study performed in a Saudi college revealed that, despite having a positive attitude toward breastfeeding, future female physicians demonstrated a low level of knowledge of the subject, along with several misconceptions [15].

Training packages and textbooks have been developed to facilitate breastfeeding training for medical students, residents and physicians [4, 16–21]. In 2009, the World Health Organization (WHO) released its ‘Infant and young child feeding: model chapter for textbooks for medical students and allied health professionals’, hereafter abbreviated as the ‘WHO infant feeding chapter’ [22]. In nine sessions, the chapter covers the importance of breastfeeding, the basic science of lactation, maternal and infant problems, and the provision of breastfeeding support through maternity services and the community.

Expert opinion on the content of breastfeeding teaching materials designed for medical students is an area of interest of this study, the objectives of which were: 1) to gather the opinions of medical teachers (MTs) on current breastfeeding medical education, and 2) to determine their views regarding the application of the WHO infant feeding chapter in the curriculum of Saudi medical colleges.

## Methods

### Study design

This study was conducted using a mixed-method approach (i.e., qualitative and quantitative methods).

### Study setting

In 2014, there were four governmental medical colleges in Riyadh, Saudi Arabia. To ensure participants were experienced in the whole curriculum (i.e., preclinical and clinical phases), only the three colleges with successfully graduated medical students were included in the study. Ethical approvals were obtained from the selected colleges.

### Study participants

Lists of MTs who were involved in the curriculum at the target colleges were obtained. A diversity of clinical and basic medical science faculty members, as well as experts in medical education, was included. In order to achieve a rich insight, clinical teachers specializing in obstetrics, pediatrics, and family medicine were the main focus of the study. The MTs were invited to participate in the study through e-mail communication, and informed consent was obtained. Study materials were sent as electronic files to those who agreed to participate. Eighteen participants were recruited initially, with an equal number from each college. The sample size was similar to that used in quantitative research studies such as Delphi technique, which usually require between 8 and 12 participants to reach data saturation [23].

### Materials

Materials used in the study included the participation invitation letter, the WHO infant feeding chapter [22], the Saudi code for breast milk substitutes [24] and the questionnaire for each of the three consecutive study rounds.

### Data collection and analysis

#### Round 1

An open-ended questionnaire containing three major sections was used as a data collection tool for the participants in round 1 of the study. This was constructed based on a literature review and the contents of the chapter being evaluated, followed by a meeting of the researchers to incorporate real-world experience. The first questionnaire section focused on the general opinion of the participants on current breastfeeding education in medical colleges. The second section assessed participants’ opinions on the WHO infant feeding chapter sessions as a tool for teaching medical students. The third section addressed the applicability of the chapter to breastfeeding education in a Saudi setting. A pilot study was conducted and the questions were modified to avoid repetition and ensure understanding of the ideas. The duration of the interview was also assessed.

Scheduled one-to-one interviews (i.e., face-to-face in the workplace or via Skype) were conducted by three interviewers (female main investigator, male co-author and female Master’s student in medical education) who were all medical doctors and trained by the main investigator. Each interviewer communicated directly with participants from a single college to establish relationships and was made aware of the research goals before scheduling interviews, each of which lasted 45–60 min. Interviews were recorded digitally and transcribed verbatim. All transcripts were read in their entirety by the principal investigator and the content was not shared with the participants. This process was repeated for each interview to capture the full breadth and diversity of the teachers’ views and experiences. Thematic analysis was performed anonymously by the main researcher. Data saturation was obtained.

#### Round 2

Utilizing the analyzed data obtained in the first round of the study, the data collection form was modified to a self-administered, closed-ended questionnaire using a 5-point Likert scale for all the sections. The questions were rewritten to incorporate the themes identified in the first round. A consensus was considered to have been reached when greater than 70% agreement was achieved within the expert team. The numbers and percentages of the individual MT responses were calculated. The response data for each of the chapter’s sessions were merged to a 3-point Likert agreement/disagreement scale to obtain an overall result.

#### Round 3

A closed-ended questionnaire was developed that focused on controversial aspects of the results of round 2. All participants were made aware of the areas of disagreement and were invited to review their opinions and explain the reasons for their responses. The percentages of MT responses were calculated. As in round 2, a consensus was considered to have been reached when greater than 70% agreement was achieved within the expert team.

## Results

### Participants

Twelve MTs participated in the study and the overall response rate was 66.7%; their characteristics are presented in Table 1. Three rounds of investigation were conducted with all participants. Data were analyzed at the end of each stage of the study and the results were used to construct the subsequent round. The response rates of the participants in each round were 100.0, 83.3 and 66.6%, respectively.

**Table 1:** Participant characteristics (*N* = 12)

Characteristics		Number
Sex	Male	6
	Female	6
Nationality	Saudi	9
	Non-Saudi	3
Specialty^a^	Medical education	(3)
	Clinical medical teacher	Pediatrics (2)
		Obstetrics and gynecology (3)
		Family medicine (4)
	Basic science medical teacher	Anatomy (1) Physiology (1)
Academic position	Lecturer	1
	Assistant professor	8
	Associate professor/professor	3
Medical college	Al-Imam Mohammad Ibn Saud Islamic University	4
	King Saud Bin Abdulaziz University for Health Sciences	4
	King Saud University	4

^a^Some medical teachers have qualification in medical education beside their clinical specialty

### Data analysis

#### Round 1

The areas of breastfeeding education in medical colleges that were discussed with the participants were summarized as those relating to the student (competency and the assessment), the curriculum (integrated, unified to both gender, culturally based content, unique to medical student, rich in content) and the teacher (expert and interprofessional). The themes and their representative quotations are reported in Table 2. There was a disagreement in terms of participant opinion regarding the need for a unique curriculum for medical students, i.e. one that is different from the curriculum used by other healthcare professionals. In general, there was a consensus among the participants regarding the different sessions of the WHO infant feeding chapter, to varying degrees, that should be included in the curriculum; however, the inclusion of the ‘breastfeeding position and attachment’ and ‘reporting breastfeeding observation’ sessions was viewed by some of the participants as unnecessary parts of the curriculum. The potential health risks of milk formula were an aspect that was not covered in the chapter under evaluation and the addition of this topic was suggested by one MT (a pediatrician).

**Table 2:** Themes and quotations relating to breastfeeding medicine education for medical students: medical teachers’ views

Themes	Quotations
The competence of the medical students in breastfeeding medicine is not optimal.	‘Actually, breastfeeding education in our college is very limited, which does not facilitate good support for breastfeeding or education for mothers.’ ‘There is a lot of confusion about baby formula, about whether it is the same, or merely similar to, mother’s milk, or better than mother’s milk.’ (MT6)
Textbooks used by medical colleges are not rich in breastfeeding medicine content.	‘There is a lack of good resources on this topic in our college curriculum.’ (MT3)
	‘There is some information about breastfeeding in the general pediatrics book, but its depth is not acceptable.’ (MT6)
Low or variable level of experience of medical teachers in breastfeeding medicine.	‘If the information about breastfeeding in the textbook is not present, the teachers are also deprived of the knowledge in breastfeeding as they themselves were not taught about it properly.’ (MT5)
	‘I don’t think that medical teachers have enough experience in lactation. Even I, an obstetrician for the last 20 years, have learned so many things in this book.’ (MT1)
	The pediatrician said, ‘I may be interested in some part of it. For example, I would like to be an expert in teaching the students in the clinical phase about the components of breast milk, because I need them to know by heart how to compare breast milk with formula.’ (MT7)
One curriculum on breastfeeding medicine should be established, regardless of the gender of the medical student.	‘We need the same curriculum for both male and female students because they are professional, and they are exposed to the same situations with the same patients.’ (MT2)
	‘It should be equal, even though the female students may someday breastfeed themselves.’ (MT5)
Out of respect for the culture, practical clinical training should be increased for female medical students.	‘Female Saudi medical students should be taught in more depth than males because they are involved more with female care. They can give care with no barriers of sex. She is also a female, and she is a mother or will be a mother in the future.’ (MT6)
Adoption of a breastfeeding teaching module for medical students with customization to fit the culture and the medical college’s curriculum is recommended.	‘If there is a ready module that can be customized, it would be good.’ (MT7)
	‘I think we can adapt the WHO module and incorporate it in our curriculum.’ (MT5)
Breastfeeding medicine education should be integrated throughout the years of the medical college’s curriculum. A short, a focused breastfeeding course within one of the major blocks, such as pediatrics or gynecology, should be established.	‘I think breastfeeding education could be scattered, with a condensed study that should be given in a small, short period of time. It is a lot for one block.’ (MT7)
	‘Breastfeeding is a great topic and a very large topic, and to have a strong foundation of information, you should present it in stages.’ (MT6)
Assessment (examination) of medical students in breastfeeding is a must.	‘Assessment of medical students in breastfeeding is a definite need as long as it is taught.’ (MT7)
	‘Medical students should pass breastfeeding medicine before they become fully licensed.’ (MT5)
A unique curriculum for medical students that differs from that of other health professions is a controversial subject.	‘Every health professional has a role and the physician’s curriculum definitely is different.’ (MT4)
	‘Why not? Health professionals should have the same breastfeeding education. Anybody who has weak information will destroy the medical practice.’ (MT6)

To adapt the WHO chapter to Saudi culture, the participants recommended customization in aspects such as breastfeeding policy and practice in the country, prevalence of breastfeeding and complementary diet. In addition, the incorporation of Islamic literature on breastfeeding education was another controversial matter identified in this round. Some participants acknowledged that male students in clinical training experience difficulties practicing in maternity wards. The interviews ended with a request for the participants to provide final comments, and the following quotations were noted:

‘Breastfeeding is a neglected field.’ (MT1)

‘Actually, we need to emphasize breastfeeding for medical students because this is the theme in the world at the current time.’ (MT2)

‘The WHO infant feeding chapter is comprehensive, easy to read and well-informed.’ (MT1)

‘I do not feel that we need more than whatever information is in the WHO infant feeding chapter. I really loved it.’ (MT1)

‘I think the WHO infant feeding chapter is too much if given in one block. If we had a summary of it, it would be suitable for Saudi Arabia.’ (MT3)

‘Education regarding the risks of formula feeding should be added to the curriculum.’ (MT7)

‘We might expect some resistance to changing the curriculum in medical colleges from different points of view. A national initiative would be better than implementing the infant feeding chapter in individual medical colleges.’ (MT2)

#### Round 2

Table 3 presents the responses of the participants regarding general breastfeeding education for medical students. In total, 84.5% of the respondents agreed on the suitability of the WHO infant feeding chapter’s contents. Table 4 presents the responses scored according to the 3-point Likert agreement/disagreement scale for each of the chapter’s sessions.

**Table 3:** Medical teachers’ opinions on breastfeeding education for medical students according to a 5-point Likert scale

Themes	Number of opinions (%)				
	Strongly disagree	Disagree	Neutral	Agree	Strongly agree
The competence of medical students in breastfeeding medicine is not optimal.	0 (0)	2 (20)	0 (0)	5 (50)	3 (30)
Textbooks used by medical colleges are not rich in breastfeeding medicine content.	0 (0)	1 (10)	0 (0)	7 (70)	2 (20)
Low or variable level of experience of medical teachers in breastfeeding medicine.	0 (0)	0 (0)	2 (20)	5 (50)	3 (30)
One curriculum on breastfeeding medicine should be established, regardless of the gender of medical students.	0 (0)	0 (0)	1 (10)	3 (30)	6 (60)
Out of respect for the culture, practical clinical training should be increased for female medical students.	**0 (0)** ^**a**^	**2 (20)**	**3 (30)**	**4 (40)**	**1 (10)**
Adoption of a breastfeeding teaching module for medical students with customization to fit the culture and the medical college’s curriculum is recommended.	0 (0)	0 (0)	1 (10)	3 (30)	6 (60)
Breastfeeding medicine education should be integrated throughout the years of the medical college’s curriculum. A short, focused breastfeeding course within one of the major blocks, such as pediatrics or gynecology, should be incorporated.	0 (0)	0 (0)	1 (10)	5 (50)	4 (40)
Assessment (examination) of medical students in breastfeeding should be mandatory.	0 (0)	1 (10)	2 (20)	5 (50)	2 (20)
A unique curriculum for the education of medical students that differs from that used for other health professions is recommended.	0 (0)	1 (10)	2 (20)	5 (50)	2 (20)
Addition of Islamic literature on breastfeeding education is recommended.	0 (0)	1 (10)	1 (10)	5 (50)	3 (30)

^a^Numbers in bold indicate that consensus criteria (70%) were not met

**Table 4:** Medical teachers’ opinions on the suitability of the content of the WHO infant feeding chapter for Saudi medical colleges, according to a 3-point Likert scale

Sessions	Number of opinions (%)		
	Disagree	Neutral	Agree
Session 1: The importance of infant and young child feeding and recommended practices.			
Medical students should learn the importance of breastfeeding.	0 (0)	0 (0)	10 (100)
Medical students should learn the evidence- based benefits of breastfeeding and complementary feeding.	0 (0)	0 (0)	10 (100)
Medical students should learn about recommended infant and young child feeding.	0 (0)	0 (0)	10 (100)
Medical students should learn the current global practices of infant nutrition.	0 (0)	1 (10)	9 (90)
Medical students should learn about current practices in infant nutrition in Saudi Arabia.	0 (0)	0 (0)	10 (100)
Medical students should learn about global strategies for infant nutrition.	0 (0)	2 (20)	8 (80)
Session 2: The physiological basis of breastfeeding.			
Medical students should learn about growth and development in relation to breastfeeding.	0 (0)	0 (0)	10 (100)
Medical students should learn about breast milk composition.	0 (0)	0 (0)	10 (100)
Medical students should learn about the anatomy of the breast.	0 (0)	1 (10)	9 (90)
Medical students should learn about hormonal control of milk production.	0 (0)	0 (0)	10 (100)
Medical students should not learn about position and attachment.	**5 (50)** ^**a**^	**2 (20)**	**3 (30)**
Medical students should learn about the composition of infant formula.	0 (0)	1 (10)	9 (90)
Medical students should learn the indications for breast milk substitutes.	0 (0)	0 (0)	10 (100)
Session 3: Complementary feeding.			
Medical students should learn about complementary food, depending on food available locally.	0 (0)	1 (10)	9 (90)
Medical students should learn about the micronutrients needed by infants and their deficiency in infancy.	0 (0)	1 (10)	9 (90)
Session 4: Management and support of infant feeding in maternity facilities.			
Medical students should be taught about breastfeeding support in maternity hospitals.	**0 (0)** ^**a**^	**4 (40)**	**6 (60)**
Male medical students should have other teaching methods, such as simulations or videos, to build their breastfeeding support skills.	0 (0)	1 (10)	9 (90)
Medical students should learn the concept of the baby-friendly hospital initiative.	0 (0)	3 (30)	7 (70)
Medical students should learn in maternity facilities that are accredited as baby-friendly.	**0 (0)** ^**a**^	**4 (40)**	**6 (60)**
Teaching medical students in maternity services that are not supportive of lactation will weaken their knowledge and their attitude toward breastfeeding.	2 (20)	1 (10)	7 (70)
Session 5: Continuing support for infant and young child feeding.			
Medical students should learn about the continuous support of breastfeeding after delivery and hospital discharge to the community.	0 (0)	2 (20)	8 (80)
Medical students should learn about the skills needed for counseling in breastfeeding.	0 (0)	0 (0)	10 (100)
Medical students are not expected to have skills related to reporting of breastfeeding observation.	**4 (40)** ^**a**^	**2 (20)**	**4 (40)**
Session 6: Appropriate feeding in exceptionally difficult circumstances.			
Medical students should learn about special situations demanding urgent referral of infants for nutritional issues.	0 (0)	0 (0)	10 (100)
Medical students should understand the issues related to feeding low birth weight infants.	1 (10)	0 (0)	9 (90)
Medical students should learn how to deal with malnourished children.	1 (10)	1 (10)	8 (80)
Medical students are not expected to learn about management of infant nutrition during disasters, such as war.	**5 (50)** ^**a**^	**3 (30)**	**2 (20)**
Session 7: Management of breast conditions and other breastfeeding difficulties.			
Medical students should learn about relactation.	0 (0)	3 (30)	7 (70)
Medical students should learn about feeding infants with HIV-positive mothers.	0 (0)	0 (0)	10 (100)
Medical students should learn about breast engorgement and nipple conditions.	0 (0)	0 (0)	10 (100)
Medical students should learn about low milk supply.	1 (10)	0 (0)	9 (90)
Medical students should learn about breast refusal.	1 (10)	0 (0)	9 (90)
Medical students should learn about feeding twins.	1 (10)	1 (10)	8 (80)
Medical students should learn about feeding infants born by Cesarean section.	0 (0)	1 (10)	9 (90)
Medical students should understand the special feeding needs of jaundiced babies.	0 (0)	0 (0)	10 (100)
Medical students should understand the feeding needs of babies with congenital anomalies, such as Down Syndrome and cleft palate.	0 (0)	1 (10)	9 (90)
Session 8: Mothers’ health.			
Medical students should learn about maternal conditions that can have an impact on lactation.	0 (0)	0 (0)	10 (100)
Medical students should learn about the maternal diet during lactation.	0 (0)	0 (0)	10 (100)
Medical students should learn about medication use during lactation.	0 (0)	0 (0)	10 (100)
Medical students should learn about family planning during lactation.	0 (0)	0 (0)	10 (100)
Session 9: Policy, health system and community actions.			
Medical students should understand the national policies that support breastfeeding, such as maternity leave rules.	2 (20)	1 (10)	7 (70)
Medical students should understand national standards for breast milk substitutes.	**2 (20)** ^**a**^	**2 (20)**	**6 (60)**
Session 10: Boxes, tables and pictures.			
The boxes in the WHO book are suitable for medical students.	0 (0)	1 (10)	9 (90)
The tables in the WHO book are suitable for medical students.	0 (0)	1 (10)	9 (90)
The pictures in the WHO book are suitable for medical students.	0 (0)	2 (20)	8 (80)

^**a**^Numbers in bold indicate that consensus criteria (70%) were not met

#### Round 3

The participants’ opinions regarding most of the controversial areas listed in Table 4 were revised, allowing a consensus to be reached with agreement levels of 75 to 100% (teaching in maternity hospitals [75%], baby-friendly environment [75%], learning about position and attachment [100%], infant feeding during disasters [75%] and the code of breast milk substitutes [87.5%]). The reasons given by the participants for their responses were as follows:

‘The best place to teach breastfeeding is in a maternity hospital (besides primary care settings).’ (MT3)

‘Being in a maternity hospital will give better exposure to all breastfeeding techniques and problems.’ (MT10)

‘A baby-friendly environment will ensure that the student will learn and adapt the breastfeeding module.’ (MT10)

‘Accredited facilities indicate high quality of care.’ (MT3)

‘Medical students should be familiar with the normal female behavior toward her infant, of which breastfeeding is the most important aspect. He, or she, will be exposed to these norms regularly.’ (MT1)

‘I think it is better to have teaching on infant nutrition in disasters, especially with the current situation in the Arabic world.’ (MT9)

‘Disasters can occur anytime, anywhere. Therefore, students should learn medical support in disasters in case they happen.’ (MT12)

A disagreement still existed regarding additional practical clinical training for female medical students and teaching observation of breastfeeding. The reasons for their responses were as follows:

‘I believe that, with respect to Saudi culture, a new mother learning about breastfeeding is more likely to be receptive to a female practitioner than a male. Therefore, additional training for the female medical student is advisable.’ (MT6)

‘In order to respect patient privacy and choices, female students should be trained more in depth to support them.’ (MT9)

‘There should be no discrepancy in the training of medical students in terms of their gender since it is not applicable in hospital practice.’ (MT1)

‘Male and female students both will have female patients in their practice.’ (MT4)

‘Male and female students must be trained equally, as some clinical settings in rural areas have mainly male health providers, so better to train both genders in the same manner.’ (M12)

‘Regarding improving writing, thinking and other skills, it is a good opportunity for the student to master them early, because it will help them in the future while writing a patient’s report or preparing to be a speaker at conferences. Why should they lose it [the opportunity]?’ (MT11)

‘Medical students are expected to have skills to report their observations; this is part of medical training.’ (MT3)

## Discussion

The present study focused on an important public health matter: breastfeeding education as a part of medical school curricula. The motivation behind this research was the lack of a standard framework for breastfeeding education in medical schools within the cultural context of Saudi Arabia. One study that examined the content of breastfeeding curricula within the medical programs of Australian medical colleges found variability in formal and clinical training [25]. The results of the present study could be used to support medical college curriculum committees in their self-evaluation of the content and appropriate integration of a lactation syllabus.

Finding qualified experts in breastfeeding medicine in academia to participate in this study proved challenging. This deficiency appears to be common around the world due to the paucity of undergraduate and/or postgraduate fellowship programmes in this field [26]. Although breastfeeding area was within the scope of the expertise of the study participants, involvement of non-academic healthcare professionals who are qualified experts in lactation may help in the development of a national curriculum framework.

The foundation of any teaching program focuses on the teacher, the student and the curriculum [1]. Unsurprisingly, there was a consensus among the expert team regarding the existence of shortcomings in this three-pronged approach in the field of lactation education within Saudi medical colleges. One study demonstrated that physicians possess sufficient information about the protective effects of breastfeeding, although the mothers who participated in their study did not receive education from their physicians [27]. Another study suggested that the program should be taught by physicians who are qualified faculty members recognized by their peer group and certified by specialty examining boards [28]. An international board-certified lactation consultant (IBCLC) was found to be more efficient as an inter-professional educator for pediatric residents [29]. The University of Illinois has developed strategies to increase breastfeeding education in the medical school curriculum, and taught by an inter-professional team [30]. The use of inter-professional education (IPE) in undergraduate health profession programmes in some Western universities has been found to be an effective tool for improving communication and interaction between healthcare providers [31]. As such, this issue clearly deserves the attention of the medical education community.

There is literature in the field of lactation science to support further curriculum development. The WHO infant feeding chapter, which is assigned for use by medical students and other healthcare professionals, was chosen as the basis of this study. According to expert opinion, the WHO infant feeding chapter was suitable in terms of content for medical students in Saudi Arabia. Almost all its topics were considered applicable to varying degrees. The expert team agreed on the necessity of a unique curriculum for medical students, and their justification was that the curriculum of the college has its own objective for physician profession. Discordance was observed in the responses obtained in rounds 1 and 2 of the study, which related to teaching about breastfeeding position and attachment and reporting observation of breastfeeding. This interesting finding was in contrast to the guidance in the handbook for breastfeeding used by physicians, in which position and attachment and the observation of breastfeeding are considered to be part of the medical management of new mothers [4]. Thus, it appears that breastfeeding education is placed at the intersection of various health science fields, and further work is needed in this area.

The participants acknowledged that delivering the full WHO infant feeding chapter in one block would be difficult, and recommended scattering the content throughout the preclinical and clinical education phases. This recommendation was consistent with the findings of another study indicating that the approach to development of the breastfeeding and lactation curriculum for medical students is not dissimilar to that used for studying other organ systems, such as the cardiovascular system or the renal system. This conclusion was based on the fact that understanding of these systems includes aspects of anatomy, physiology, biochemistry, pharmacology, normal function, pathology and finally, clinical application in a wide range of clinical settings [28].

The MTs surveyed in this study recommended customization of the WHO infant feeding chapter to suit Saudi culture, with emphasis on the prevalence of breastfeeding in Saudi Arabia, social context, Islamic perspective, complementary food and national policies regarding infant nutrition. In the second round, there were conflicting opinions regarding teaching the national code of breastfeeding substitutes, while in the third round, there was agreement regarding the appropriateness of teaching this subject ‘in brief’. Although the Saudi code was circulated electronically to all participants, the reasons given in support of their opinions were poor in content. From a medical ethics standpoint, it is important to inform medical students, as future healthcare workers, about the concept of the Saudi code [32].

Despite the advent of medical simulation, the expert team was supportive of clinical teaching of breastfeeding in maternity wards and, preferably, in a baby-friendly environment. Three hospitals are designated as baby-friendly in Riyadh; however, the feasibility of training all students in these hospitals or the availability of designated baby-friendly hospitals in other cities represents a potential concern. Interestingly, the MTs recommended that the same curriculum should be implemented regardless of the gender of the medical students. However, there was a difference of opinion about increased clinical training for females, whether they are mothers or simply health professionals, as opposed to male future physicians in Saudi Arabia. Gender differences in medical education are a hot topic in some fields, such as breastfeeding, obstetrics and gynecology [33], and research shows that female medical students are also in need of breastfeeding education [34].

This study was conducted in a very small sample of MTs and may be limited by an inherent deficiency of qualified experts in lactation in the medical education field; this may have contributed to response bias. The attainment of a consensus may be affected by a significant loss of participants in round 3. In addition, the participating colleges, all located in one city, may not reflect the opinions of MTs in Saudi Arabia as a whole. These limitations should be considered during the development of a Saudi breastfeeding medical education framework.

## Conclusions

The medical education community should address education of lactation management in more depth than it currently does during undergraduate medical program. A national framework for a breastfeeding curriculum for implementation in Saudi medical colleges is urgently needed. The use of resources such as the WHO infant feeding chapter in a physician-specific breastfeeding curriculum is highly recommended. Finally, developing specialized clinical training in breastfeeding for both male and female medical students is an important area for future research.

## Abbreviations

MT: Medical teacher
WHO infant feeding chapter: World Health Organization ‘Infant and young child feeding: model chapter for textbooks for medical students and allied health professionals’ [22]
WHO: World Health Organization
